# Electroacupuncture at ST36 Improve the Gastric Motility by Affecting Neurotransmitters in the Enteric Nervous System in Type 2 Diabetic Rats

**DOI:** 10.1155/2021/6666323

**Published:** 2021-06-16

**Authors:** Xu Han, Xiaoyan Chen, Xuan Wang, Meirong Gong, Mengjiang Lu, Zhi Yu, Bin Xu, Jinhong Yuan

**Affiliations:** ^1^Key Laboratory of Acupuncture and Medicine Research of Ministry of Education, Nanjing University of Traditional Chinese Medicine, Nanjing 210023, Jiangsu, China; ^2^Nanjing University of Traditional Chinese Medicine, Nanjing 210023, Jiangsu, China

## Abstract

Electroacupuncture (EA) can effectively relieve hyperglycemia and gastric emptying disorders in diabetic gastroparesis (DGP). However, the effect of EA on type 2 diabetes mellitus (T2DM) gastroparesis and its mechanism in the enteric nervous system (ENS) are rarely studied. We investigated the therapeutic effect of EA at ST36 and its effect on the main inhibitory and excitatory neurotransmitters in the ENS in DGP rats. Male Sprague-Dawley (SD) rats were fed a high-fat diet for 2 weeks and injected with streptozotocin (STZ) at 35 mg/kg to induce T2DM. T2DM rats were divided into the diabetic mellitus (DM) group and the EA group. The control (CON) group comprised normal rats without any intervention. EA treatment was started 6 weeks after the induction of DM and continued for 5 weeks. The body weight and food intake of the rats were recorded every week. Blood glucose, insulin, glucose tolerance, gastric emptying, and antral motility were measured after treatment. The expression of protein gene product 9.5 (PGP9.5), neuronal nitric oxide synthase (nNOS), and choline acetyltransferase (ChAT) in gastric antrum were quantified by western blotting and quantitative real-time reverse transcription polymerase chain reaction (qRT-PCR). The T2DM gastroparesis model was successfully established. EA treatment reduced the body weight, food intake, and blood glucose; improved glucose intolerance and insulin resistance; increased the gastric emptying rate, the mean antral pressure, and the amplitude of antral motility; and decreased the frequency of antral motility compared with those in the DM group. EA treatment increased the expression level of nNOS, ChAT, and PGP9.5 proteins, and nNOS and ChAT mRNA. The results suggested that EA at ST36 could ameliorate DGP, partly restore the damage to general neurons, and increase nNOS and ChAT in the gastric antrum. EA improved DGP partly via reducing the loss of inhibitory and excitatory neurotransmitters in the ENS.

## 1. Introduction

Gastroparesis is defined as a syndrome of delayed gastric emptying without mechanical obstruction. The main symptoms include early satiety, postprandial fullness, nausea, vomiting, abdominal distension, and abdominal pain [1]. Diabetic gastroenteropathy is the most common complication in patients with long-term DM. The most common complication of diabetes in the gastrointestinal tract is delayed gastric emptying, which is defined as DGP [2]. Although DGP seems to be more common in type 1 diabetes mellitus (T1DM) than in T2DM, the increased prevalence of T2DM leads to a greater number of patients with type 2 diabetic gastroparesis [1].

Conventional western medicine treats gastroparesis using prokinetic drugs, gastric pacemaker, and surgery; however, the side effects, such as extravertebral symptoms, arrhythmia, infection, and malnutrition, are difficult to avoid. Acupuncture, as a complementary and alternative treatment technology, can effectively improve gastrointestinal peristalsis and accelerate gastric emptying [3]. Acupuncture can improve gastrointestinal symptoms in patients with various diseases. A randomized crossover clinical study evaluated the short-term effects of acupuncture on patients with DGP. The results showed that acupuncture for one week could significantly reduce gastric retention and alleviate the symptoms in patients with DGP [4].

The pathological mechanisms of DGP include autonomic neuropathy, enteric neuropathy, abnormal interstitial cells of Cajal (ICC), and acute fluctuation of blood glucose [1]. An abnormal ENS plays an important role in the pathogenesis of DGP. One of the main components of the ENS is the myenteric plexus, which is a neural network that coordinates gastric motility. The myenteric plexus includes excitatory and inhibitory motor neurons, as well as primary afferent neurons and intermediate neurons. Cholinergic neurons are excitatory motor neurons that induce muscle contraction by releasing acetylcholine (ACh), synthesized by ChAT. Nitrergic neurons, which are inhibitory neurons, relax muscle tissue by releasing nitric oxide (NO) synthesized by nNOS (also known as NOS1) [3, 5, 6]. Changes of ChAT and nNOS levels might affect the control of gastric motility and lead to gastric rhythm disorders. Human and animal studies have shown that diabetes can lead to decreased levels of nNOS and ChAT in the gastrointestinal tract, and these losses are more reversible than initially thought [7]. The loss of nNOS in the ENS can lead to impaired inhibitory input, resulting in dysregulation of gastric antrum contraction and delayed gastric emptying [8]. Studies have shown that EA can improve DGP in rats by regulating the abnormality of the autonomic nervous system or ICC [9–11]. To date, there has been little research on the mechanism of ENS in DGP rats treated with EA.

In the present study, we aimed to establish a rat model of T2DM gastroparesis to explore the therapeutic effect of EA on DGP, and to partially explore the mechanism of neurotransmitters in the ENS.

## 2. Materials and Methods

### 2.1. Animals and Induction of T2DM

Male SD rats, weighing 180–200 g, were obtained from the experimental animal center of Nanjing University of Traditional Chinese Medicine (Nanjing, China). The rats were housed in a controlled environment (12 h light/dark cycle, 22 ± 2°C, humidity 50 ± 10%), with free access to water and feed. The animals were raised adaptively for one week before the experiment, during which their general health conditions were observed. The protocols for the use and care of animals were approved by the ethics committee of Nanjing University of traditional Chinese Medicine (Approval Numbers: 201910A026; 202004A008). Laboratory animals were raised according to internationally recognized principles for the use of laboratory animals.

**Figure 1:**
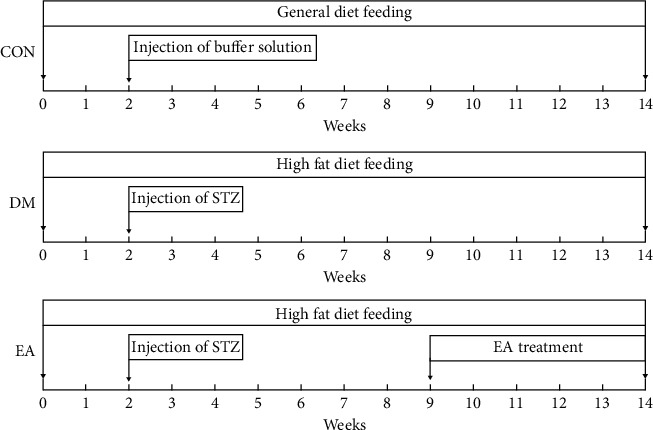
Experimental design and treatment protocol.

In order to minimize potential confounders, the random number table was used throughout the experiment. After adapting to the environment, the rats were randomly divided into the CON group and the model group. The CON group was fed a normal diet, and the model group was fed a high-fat diet (58% fat, 25% protein, and 17% carbohydrate, as a percentage of total kcal). After 2 weeks, all animals were fasted for 14 hours. The rats in the model group were intraperitoneally injected with (STZ) (Sigma, St. Louis, MO, USA) dissolved in 0.1 M citric acid/sodium citrate buffer (pH 4.5), and the dose was 35 mg/kg. The rats in the CON group were given 3.5 mL/kg citric acid/sodium citrate buffer. Blood glucose was measured using a portable glucometer (Roche, Indianapolis, IN, USA). The animals with a nonfasting blood glucose (NFBG) level > 16.7 mmol/L were determined as having T2DM [12]. Six weeks after T2DM was established, the diabetic rats were divided into the DM group and the EA group. To maintain the state of diabetes, the animals were fed the high-fat diet until the end of the experiment. Euthanasia should be performed when the animal is too weak to eat (intraperitoneal injection of 25% uratan, 10 ml/kg) to minimize the animal's suffering. The experimental design is shown in Figure 1.

### 2.2. EA Intervention

**Figure 2:**
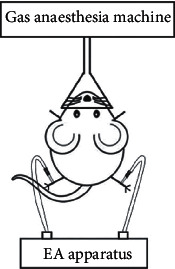
The diagram of EA intervention in rats.

Acupuncture point ST36 (Zusanli) is located in the posterolateral aspect of the knee joint, about 5 mm below the capitulum fibulae [13]. The needle was connected to electric acupuncture apparatus (Hanshi, Nanjing Jisheng Medical Technology Co., Ltd., Nanjing, China), and the parameters were set as 2 mA, 2/15 Hz. Rats in the EA group were treated with ST36 on both sides from the 9th week to the 14th week of the experiment (20 minutes per day, 6 days a week). The diagram of EA intervention in rats is shown in Figure 2.

### 2.3. Blood Sampling and Intraperitoneal Glucose Tolerance Test (IGTT)

The animals were deprived of food overnight, after which blood samples were collected from the orbit and centrifuged at 231rcf for 15 minutes. After that, the serum was isolated and stored at −80°C to measure insulin levels, which was performed by Nanjing Jiancheng Bioengineering Institute. Rats were injected intraperitoneally with 50% glucose (2 g/kg). The blood glucose was measured using a portable glucometer (Roche) at 0, 30, 60, 90, and 120 minutes after glucose injection [14].

### 2.4. Ultrasonic Examination

According to a previous study [15], the animals' abdomens were shaved before the experiment, and the rats were given 2 mL of a semisolid test meal by gavage and then anesthetized by isoflurane inhalation immediately. The gastric cross-sectional area was recorded at 0 min and 20 min after taking the test meal. The area was calculated using the built-in calculation program. The gastric emptying rate = [gastric area (0 min) − gastric area (20 min)]/gastric area (0 min)*∗*100%.

### 2.5. Phenolic Red Test

The rats were deprived of food and drinking water 19 hours before the test, and no water was allowed 1 hour before the experiment. Rats were given an experimental meal (0.05% w/v phenol red in 1.5% w/v aqueous hydroxyethyl cellulose solution, 1.5 mL p.o. per rat), after which the animals were killed by intraperitoneal injection of 25% urethane (10 mL/kg). The stomach was removed, and its contents emptied into 40 mL of 0.1 M NaOH. One milliliter of this mixture was added into 2 mL 7.4% w/v trichloroacetic acid solution to precipitate the protein. Then, the mixture was centrifuged (231 rcf, 15 min), and 2 mL of the supernatant was mixed with 1 mL of 1 M NaOH, and the absorbance of the sample was measured at 560 nm using a spectrophotometer (actual measured value). In addition, 1.5 mL of 0.05% w/v phenol red in 1.5% w/v aqueous hydroxyethyl cellulose solution was taken, and the absorbance (standard value) was measured after the same operation. The gastric emptying rate = (1 − the actual measured value/standard value)*∗*100% [16].

### 2.6. Gastric Antral Motility Detection Surgery

At the end of EA treatment, the CON group and the DM group were tested for antral motility using balloon detection. The rats were fasted overnight and then anesthetized by intraperitoneal injection of 25% urethane (5 mL/kg). The rats were placed on the experimental platform, and a 1 cm transverse incision was made in the abdominal xiphoid process. The duodenum was located, and a small incision was made at 0.5 cm away from the pylorus. The balloon manometry probe was placed into the gastric antrum, and the pressure signals were input into the physiological signal acquisition system. After the operation, the rat was left for at least 1 h for the pressure waveform to become stable and thus avoid stress. The average pressure of the gastric antrum and the amplitude and frequency of gastric antral motility were measured.

### 2.7. Tissue Harvesting and Western Blotting Analysis

After the animals were euthanized, the antrum was collected and stored immediately at −80°C. Total protein was extracted from gastric antrum homogenate, and the concentration of protein was determined using an enzyme-linked immunosorbent assay at 562 nm. The protein sample was boiled, and then equivalent amounts of protein were subjected to sodium dodecyl sulfate-polyacrylamide gel electrophoresis (SDS-PAGE) using 8% or 12% gels. The proteins were transferred onto a polyvinylidene fluoride (PVDF) membrane. TBS-T buffer (Tris buffer saline, 0.1% Tween) containing 5% BSA was used to block the membrane at room temperature for 60 minutes. Then, the membrane was incubated with the primary antibodies at 4°C overnight. Horseradish peroxidase (HRP) conjugated secondary antibodies were then incubated with the membranes at room temperature for 60 minutes. The gray values of the immunoreactive protein bands were quantified using ImageJ software (NIH, Bethesda, MD, USA). The antibodies used included anti-PGP9.5 (rabbit, 1 : 1000, Abcam, Cambridge, UK), anti-nNOS (rabbit, 1 : 1000, Abcam), anti-ChAT (rabbit 1 : 1000, Abcam), anti-GAPDH (1 : 1000, Cell signaling Technology, Danvers, MA, USA), and anti-Vinculin (1 : 2000, Abcam, Cambridge, UK) antibodies.

### 2.8. QRT-PCR

After the animals were euthanized, the antrum was collected and stored immediately at −80°C. Total RNA was extracted from approximately 100 mg of tissue using the TRIzol reagent (Invitrogen, Carlsbad, CA, USA), and 1 *μ*g of the RNA samples was reverse-transcribed according to the manufacturer's instructions (ES Science, Shanghai, China). Amplification of multiple samples was conducted using SYBR Green (ES Science). Each reaction mixture (20 *μ*L) contained 0.4 *μ*L of forward primer, 0.4 *μ*L of reverse primer, 2 *μ*L of cDNA, 10 *μ*L of SYBR Green qPCR Mix, and 7.2 *μ*L of ddH_2_O. qPCR was performed using a Mx3005P™ QPCR System (Stratagene, San Diego, CA, USA). The PCR protocol was as follows: 40 cycles of amplification for 10 seconds at 95°C and 60 seconds at 60°C. The sequences of the primers used are shown in Table 1.

### 2.9. Statistical Analysis

SPSS 22.0 software (IBM Corp., Armonk, NY, USA) and GraphPad Prism 8.0 (GraphPad Inc., La Holla, CA, USA) were used for data analysis. Values are presented as the mean ± SEM. A *t*-test was used to compare data between the two groups, and one-way ANOVA was used for comparisons among more than two groups. *P* < 0.05 indicated statistical significance.

## 3. Results

### 3.1. Effects of EA on Body Weight and Food Intake

**Figure 3:**
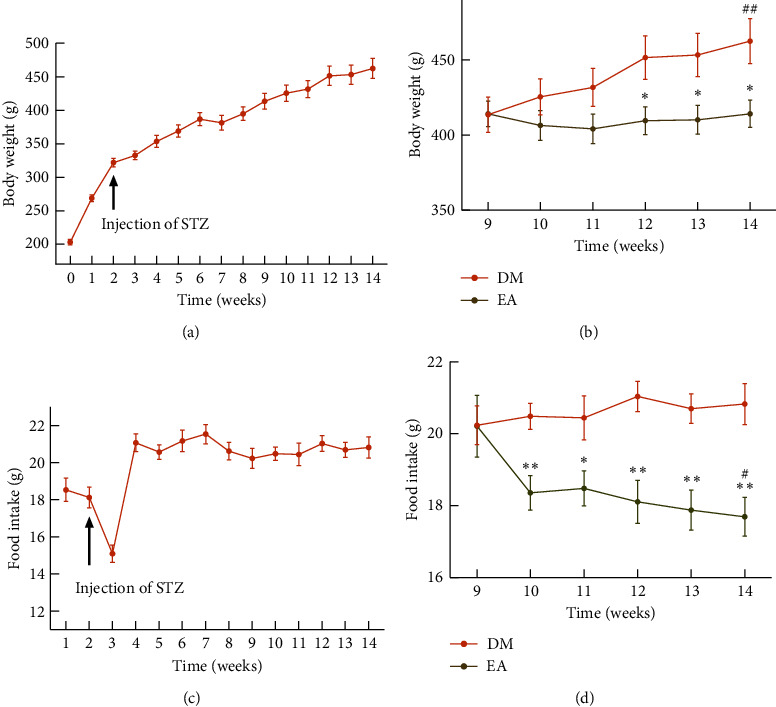
(a) The body weight of the DM group from week 0 to the 14th week (*n* = 8). (b) The body weight of the DM group and the EA group from the 9th week to the 14th week (*n* = 8). ^*∗*^*P* < 0.05 EA *vs*. DM. ^##^*P* < 0.01 the 14th week vs. the 9th week. (c) The daily food intake was recorded in the DM group from the first week to the 14th week (*n* = 6). (d) The daily food intake of rats from the 9th week to the 14th week (*n* = 6). ^*∗*^*P* < 0.05, ^*∗*^*P* < 0.01 EA *vs*. DM. ^#^*P* < 0.05 the 14th week (v) s. the 9th week. DM, the diabetes mellitus group; EA, the electroacupuncture group.

Because the rats in the CON group were fed a conventional maintenance diet, and the rats in the DM group were fed a high-fat diet, the differences in body weight and food intake between the two groups were not compared. Although the rate of increase slowed after STZ injection, the rats in the DM group continued to gain weight from the first week to the 14th week (Figure 3(a)). In the 14th week, the body weight of the rats in the DM group increased (*P* < 0.01), while that in the rats in the EA group showed no change, indicating that EA could reduce the weight of DM rats (Figure 3(b)). The food intake of the rats in the DM group decreased sharply after the injection of STZ and then increased sharply in the 4th week, after which it remained relatively stable (Figure 3(c)). In the 14th week, the food intake of the rats in the EA group decreased (*P* < 0.05), and that in the rats in the DM group did not change (*P* > 0.05); thus, EA treatment reduced the food intake of the DM rats (*P* < 0.05, Figure 3(d)).

### 3.2. Effect of EA on Blood Glucose

**Figure 4:**
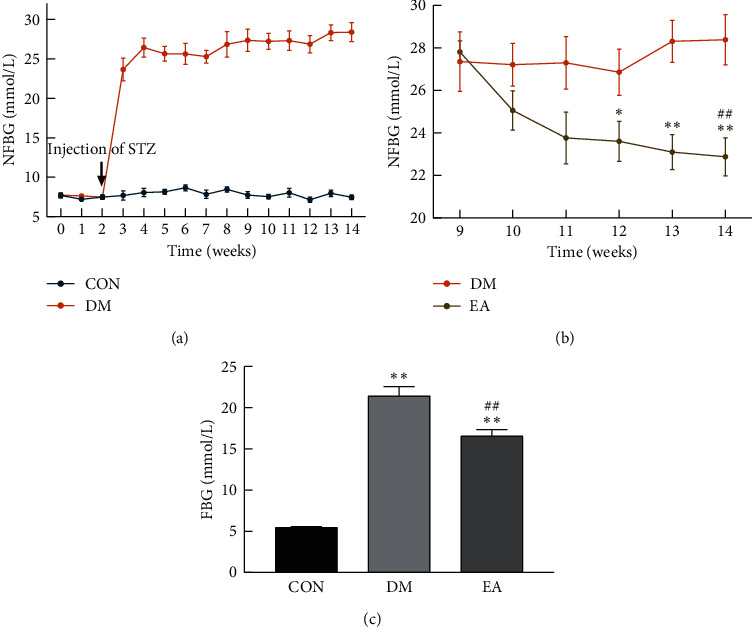
(a) The NFBG of the CON group and the DM group from week 0 to the 14th week (*n* = 8). (b) The NFBG of the DM group and the EA group from the 9th week to the 14th week (*n* = 8). ^*∗*^*P* < 0.05, ^*∗∗*^*P* < 0.01 EA *vs*. DM. ^##^*P* < 0.01 the 14th week *vs*. the 9th week. (c) The FBG in each group from the 9th week to the 14th week (*n* = 8). ^*∗∗*^*P* < 0.01*vs*. CON. ^##^*P* < 0.01*vs*. DM. CON, the control group; DM, the diabetes mellitus group; EA, the electroacupuncture group; NFBG, nonfasting blood glucose; FBG, fasting blood glucose.

Throughout the experiment, the NFBG in the rats in the CON group was always within the normal range. The NFBG of the rats in the DM group increased rapidly after the injection of STZ and remained high until the end of the experiment (Figure 4(a)). In the 14th week, the NFBG of the rats in the EA group decreased significantly (*P* < 0.01), and that in the rats in the DM group did not change (*P* < 0.05); thus, EA treatment could decrease the NFBG levels of the DM rats (*P* < 0.05, Figure 4(b)). The fasting blood glucose (FBG) of the rats in the DM group increased (*P* < 0.01), and EA treatment reduced the FBG levels (*P* < 0.01); however, they were still higher than those of the rats in the CON group (*P* < 0.01, Figure 4(c)).

### 3.3. Effects of EA on the Insulin Level, HOMA-IR, and Glucose Tolerance

**Figure 5:**
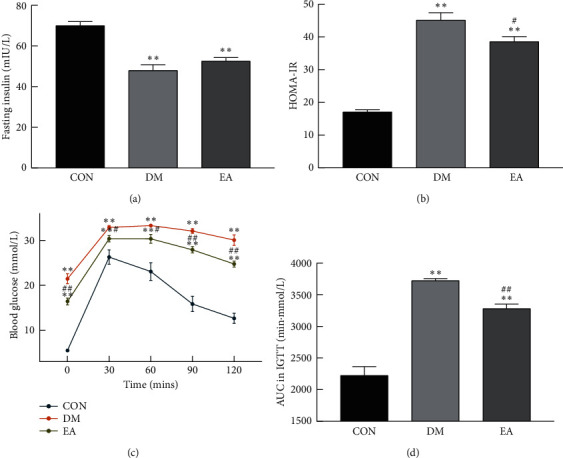
(a) The fasting insulin in each group in the 14th week (*n* = 8). (b) The HOMA-IR in each group in the 14th week (*n* = 8). (c) The blood glucose at different time points in IGTT (*n* = 8). (d) The AUC in IGTT was calculated according to figure (c) in the 14th week (*n* = 8). ^*∗*^*P* < 0.05, ^*∗∗*^*P* < 0.01 vs. CON. ^#^*P* < 0.05, ^##^*P* < 0.01 vs. DM. CON, the control group; DM, the diabetes mellitus group; EA, the electroacupuncture group; HOMA-IR, homeostatic model assessment of insulin resistance; IGTT, intraperitoneal glucose tolerance test.

At the end of treatment, the insulin level of the rats in the DM group decreased compared with that in the rats in the CON group (*P* < 0.01), and there was no difference between the rats in the DM group and those in the EA group (*P* < 0.05, Figure 5(a)). Compared with the rats in the CON group, the homeostatic model assessment of insulin resistance (HOMA-IR) (Figure 5(b)) and area under curve (AUC) in the IGTT (Figure 5(d)) of the rats in the DM group increased (*P* < 0.01), and EA decreased the HOMA-IR and AUC in the IGTT of the DM rats (*P* < 0.05). The IGTT showed that the blood glucose level of the rats in the DM group increased at all time points (*P* < 0.01), and the EA treatment decreased the blood glucose level at all time points compared with that in the rats in the DM group (*P* < 0.05, Figure 5(c)).

### 3.4. Effect of EA on the Gastric Emptying Rate

**Figure 6:**
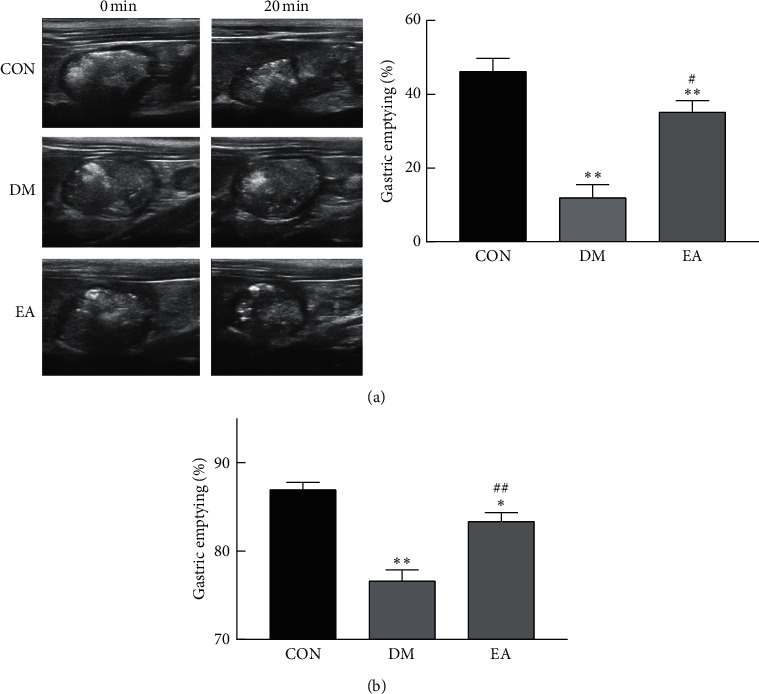
Effect of EA on the gastric emptying rate in diabetic rats. (a) The gastric emptying rate of semisolid paste as detected by ultrasound in the 14th week (*n* = 6). (b) The gastric emptying rate of liquid as detected by the phenol red test in the 14th week (*n* = 6). ^*∗*^*P* < 0.05, ^*∗∗*^*P* < 0.01*vs*. CON. ^#^*P* < 0.05, ^##^*P* < 0.01*vs*. DM. CON, the control group; DM, the diabetes mellitus group; EA, the electroacupuncture group.

In the 14th week, ultrasound examination (Figure 6(a)) and the phenol red test (Figure 6(b)) showed that the gastric emptying rate of the rats in the DM group decreased compared with that in the rats in the CON group (*P* < 0.05). EA treatment increased the gastric emptying rate of the T2DM rats (*P* < 0.05).

### 3.5. Effect of EA Treatment on Gastric Antral Motility

**Figure 7:**
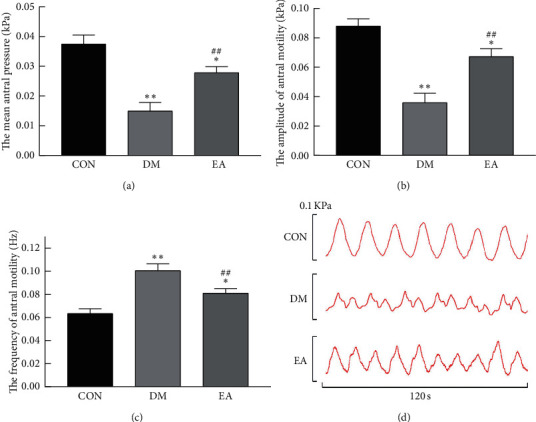
Effect of EA treatment on gastric antral motility. (a) The mean antral pressure in each group in the 14th week (*n* = 6). (b) The amplitude of gastric antral motility in each group in the 14th week (*n* = 6). (c) The frequency of gastric antral motility in each group in the 14th week (*n* = 6). (d) 120 s oscillogram of gastric antral motility in each group in the 14th week. ^*∗*^*P* < 0.05, ^*∗∗*^*P* < 0.01*vs*. CON. ^##^*P* < 0.01*vs.* DM. CON, the control group; DM, the diabetes mellitus group; EA, the electroacupuncture group.

In the 14th week, balloon gastric motility detection showed that, compared with the rats in the CON group, the mean antral pressure (Figure 7(a)) and amplitude of gastric antral motility (Figure 7(b)) in the rats in the DM group decreased, and the frequency of gastric antral motility (Figure 7(c)) increased (*P* < 0.05). EA increased the mean antral pressure and amplitude of gastric antral motility and decreased the frequency of gastric antral motility, in the T2DM rats (*P* < 0.01).  Figure 7(d) shows the waveform of gastric antral motility in each group in the 14th week.

### 3.6. Effect of EA Treatment on the Protein Expression of nNOS, ChAT, and PGP9.5 in the Gastric Antrum of T2DM Rats

**Figure 8:**
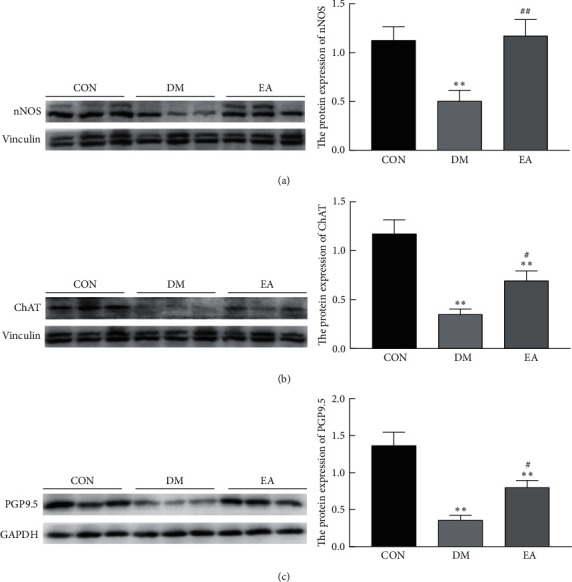
Effect of EA treatment on the protein levels of nNOS, ChAT, and PGP9.5 in the gastric antrum of T2DM rats. (a) The protein level of nNOS in the gastric antrum after in the 14th week (*n* = 6). (b) The protein level of ChAT in the gastric antrum in the 14th week (*n* = 6). (c) The protein level of PGP 9.5 in the gastric antrum in the 14th week (*n* = 6). ^*∗∗*^*P* < 0.01*vs*. CON. ^#^*P* < 0.05, ^##^*P* < 0.01*vs*. DM. CON, the control group; DM, the diabetes mellitus group; EA, the electroacupuncture group; T2DM, type 2 diabetes mellitus.

Compared with those in the rats in the CON group, the protein levels of nNOS (Figure 8(a)), ChAT (Figure 8(b)), and PGP9.5 (Figure 8(c)) in the rats in the DM group decreased (*P* < 0.01), and EA increased the protein levels of nNOS, ChAT, and PGP9.5 in the T2DM rats (*P* < 0.05).

### 3.7. Effect of EA Treatment on the Level of nNOS and ChAT mRNA in the Gastric Antrum of T2DM Rats

**Figure 9:**
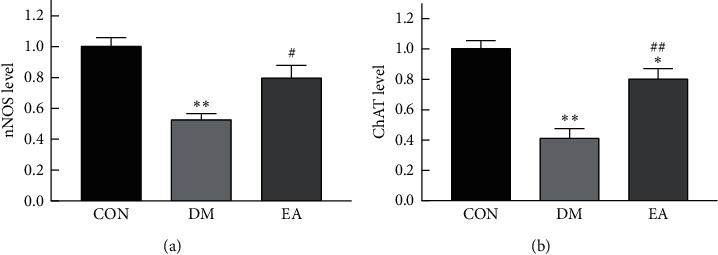
Effect of EA treatment on the level of *Nos1* and *ChAT* mRNA in gastric antrum of T2DM rats. (a) The mRNA expression of *Nos1* in the gastric antrum after in the 14th week (*n* = 6). (b) The mRNA expression of *ChAT* in the gastric antrum in the 14th week (*n* = 6). ^*∗*^*P* < 0.05, ^*∗∗*^*P* < 0.01*vs.* CON. ^#^*P* < 0.05, ^##^*P* < 0.01*vs*. DM. CON, the control group; DM, the diabetes mellitus group; EA, the electroacupuncture group; T2DM, type 2 diabetes mellitus.

Compared with that in the rats in the CON group, the mRNA level of *Nos1* (nNOS) (Figure 9(a)) and *ChAT* (Figure 9(b)) in the rats in the DM group decreased (*P* < 0.01), and EA increased the mRNA level of *Nos1* and *ChAT* in T2DM rats (*P* < 0.01).

## 4. Discussion

The prevalence of DM is increasing worldwide, which has a significant impact on the economy and on individuals. The incidence rate and mortality rate are also increasing. Most patients with DM have gastrointestinal symptoms during their course of the disease. DGP is a complex disease, and when choosing the treatment method, clinicians must carefully weigh its benefits against its adverse reactions and costs [8]. In China, acupuncture has been used for thousands of years as an effective method to treat gastrointestinal dysfunction. In recent years, acupuncture has been gradually accepted by practitioners and patients around the world. Many studies have shown that acupuncture or EA can effectively improve gastrointestinal peristalsis [17]. As a supplementary and alternative therapy, acupuncture can treat T2DM in a comprehensive and holistic way, with no side effects related to drug treatment and at low cost [18]. Therefore, acupuncture has great potential to treat type 2 diabetic gastroparesis.

For animal models of gastroparesis, most previous studies chose T1DM rather than T2DM. In fact, most patients with DGP are based on T2DM [1]. Obesity is the most common risk factor for T2DM [19]. The early stage of T2DM is mainly characterized by glucose and lipid metabolism disorder and insulin resistance, and the late stage is mainly characterized by a relative lack of insulin. The ideal model should have the above characteristics [20, 21]. In this study, T2DM was successfully induced using a high-fat diet and a low dose of STZ. Subsequent continuous high-fat diet feeding ensured the stability of the hyperglycemic state in diabetic rats, which promoted the occurrence of gastrointestinal lesions. The whole process tried to simulate the disease process of most DGP patients. The T2DM rats consumed more food and water, they became hairier, and their mental state became worse, which was in line with the previously reported characteristics of T2DM animals. EA effectively improved the general condition and glucose metabolism of the T2DM rats. Shu et al. [22] showed that EA, by reducing appetite, could improve the obesity of insulin resistant rats induced using a high-fat diet. It was speculated that EA administered at ST36 could affect the appetite of T2DM rats, reduce the intake of the high-fat diet, and reduce body weight. In the present study, EA improved blood glucose by reducing insulin resistance rather than by increasing insulin secretion. The role of acupuncture in improving IR involves multiple levels and various systems of the neuroendocrine immune network, such as regulating the levels of some related proteins in insulin target tissues (liver and skeletal muscle), inhibiting the inflammatory response of liver tissue in T2DM rats, and raising the activity of superoxide dismutase [23].

Hyperglycemia might lead to gastric rhythm disorders, antral motility disorders, and delayed gastric emptying [24]. Transabdominal ultrasonography is a noninvasive, convenient, cheap method to evaluate gastric emptying. 2-dimensional ultrasound can be used to detect the emptying of liquid or semisolid by recording the change of gastric area over time. 3-dimensional ultrasound shows more comprehensive imaging functions to provide more accurate information, such as intragastric meal distribution and gastric volume [25]. Ultrasound has been used to evaluate gastric emptying in DGP patients, and it is rarely used in animal models. In animal experiments, the phenol red test is a common method in gastric emptying detection. Because ultrasound does not cause any damage to animals, it was performed before the phenol red test. Both liquid and semisolid gastric emptying were detected, which increased the evidence to judge the model qualified. Normal gastric emptying requires the joint action of fundic relaxation and accommodation, peristaltic contractions of the antrum, and pyloric relaxation [26]. The gastric antrum is responsible for mixing and triturating the food into less than 2-3 mm during the digestive period and continuing to grind large food particles during the interdigestive period [26, 27]. The patients with long-standing DM showed antral hypomotility during the postprandial period and the fasting period [28]. The reduction of antral motility is one of the reasons for delayed gastric emptying in patients with DGP. Compared with healthy individuals, the amplitude of postprandial antral contraction is decreased, and the frequency of postprandial antral contraction is increased in patients with DGP. It is speculated that the high-frequency contraction of gastric antrum in DGP patients may be due to the imbalance between the excitatory and the inhibitory nerves firing on the antral electromechanical activity caused by vagal damage [29]. Balloon detection was performed to detect the movement of gastric antrum in the fasting state in this study, and the advantage of this method is that the waveform of gastric antrum movement can be clearly observed. Our results were similar to the above studies. It can be observed that the antral peristaltic waveform of DGP rats was not smooth and had small disordered fluctuation. A single contraction was insufficient, resulting in the completion of a peristaltic wave in a short time, which showed an increase in the frequency of antral peristalsis. The amplitude of antrum movement was small, the frequency was fast, and the mean antrum pressure was low in model rats, which indicated antral hypomotility. Few studies focus on the effect of acupuncture on gastric antral motility in DGP animal model. It is speculated that EA at ST36 might accelerate gastric emptying in T2DM rats by improving antral motility.

The ENS is an autonomous entity that exists throughout the gastrointestinal tract, and it contains sensory, motor, and interneurons that control and coordinate motility, blood flow, and secretion in the gastrointestinal tract. After the initial discovery of the extrinsic nervous system deficiency in DGP, it was found that the intrinsic nervous system was also affected. The role of ENS and its neurotransmitters in the changes of gastrointestinal function caused by DM has received increasing attention in recent years [7, 30]. Inhibitor neurotransmission in the ENS occurs through nonadrenergic noncholinergic pathway. NO is the main inhibitory neurotransmitter, with nNOS being the rate controlling enzyme in its production within ENS. Acetylcholine is the primary transmitter of excitatory muscle motor neurons in the ENS, and ChAT is responsible for ACh synthesis [31–33]. Recent studies have shown that one of the most common gastric cellular defects in gastroparesis is the loss of nNOS [1]. Clinical studies have shown that the expression of nNOS in the gastric mucosa of male patients with T2DM is decreased [34]. Animal studies showed that the numbers of NOS-immunoreactive cells in the gastric muscular plexus, and NOS activity, were significantly reduced in spontaneously diabetic rats [35]. Using immunohistochemistry, Wrzos et al. [36] observed a decrease in nNOS expression in the antral myenteric plexus of T1DM rats induced by STZ for 3 months. It was speculated that this change might be the cause of the change in gastric emptying induced by DM. Similar results were obtained in our T2DM rats, and EA could reduce the loss to some extent. Yang et al. [37] established a T1DM mouse model and found that the gastric ChAT level was reduced significantly, the myenteric cholinergic neurons and their fibers decreased significantly, and the numbers of cholinergic neurons in the gastric myenteric plexus and smooth muscle decreased from the 4th week of DM, which aggravated the progress of DGP. We obtained similar results in the rat model. The normal ENS needs a balance between the release of excitatory and inhibitory neurotransmitters [25]. The imbalance between the inhibitory and excitatory effects of neurons can lead to impaired nerve-mediated muscle responses and contribute to gastrointestinal motility dysfunction [30]. In this study, EA improved the loss of nNOS and ChAT in the gastric antrum of T2DM rats and may correct the imbalance between them, thus representing part of the mechanism, by which EA promotes gastric motility in DGP in the ENS. Diabetes is associated with changes in enteric neuronal number [30]. Pankaj et al. [38] used PGP9.5 staining to evaluate the number of nerve cell bodies and fibers in the gastric wall of patients with DM. They found that the number of nerve fibers in the round and longitudinal muscle layers was normal in sections from patients with well controlled DM, while the number of nerve fibers in patients with poorly controlled DM decreased by 40–50%. Baker et al. [39] established an ENS absorption model by local ablation of the anterior gastric antrum in mice. Delayed gastric emptying of solid and liquid was observed in the model mice, which was thought to be directly related to the loss of neurons. A loss of circular muscle enteric nerve fibers was found in the evaluation of full-thickness gastric biopsy in patients with diabetic gastroparesis [40]. Li et al. [41] established a DM rat model and, 12 weeks later, found that the neurons in the muscular layer showed depletion of axons, swelling of mitochondria, and other serious damage; in addition, the expression of PGP9.5 was significantly decreased, which is consistent with our results. Apoptosis, oxidative stress, and advanced glycation end products caused by hyperglycemia, as well as the inflammatory reaction caused by neuropeptides from ENS interacting with immune cells, can lead to neuron loss and gastrointestinal motility disorders [30, 42]. The specific protective mechanism of EA on the ENS in DGP rats remains to be explored.

It has been reported that EA can regulate the ENS in intestinal diseases. Previous studies of our team showed that EA can regulate the function of the ENS of the small intestine and proximal colon to improve intestinal dyskinesia in a mouse constipation model [43]. Du and Liu [44] showed that EA can partly restore the loss of enteric neurons in the colon of diabetic rats. Few studies have focused on the effect of EA on the ENS of the stomach. Yu et al. [45] explored the effect of EA combined with traditional Chinese medicine on the expression of substance P (SP) and nNOS in the antrum of DGP mice. However, the role of EA itself is not clear due to the inclusion of Chinese medicine in the intervention method. Our study confirmed that EA had a positive effect on the gastric motility and the ENS of gastric antrum in the animal model with DGP. We only focused on the changes of the most important neurotransmitters and the total number of enteric neurons in the gastric antrum. The changes of other neurotransmitters (such as SP, vasoactive intestinal peptide), various types of enteric neurons, the ENS in other parts of the stomach, and the effect of EA response to them are still unclear. We will conduct further investigations in the future.

## 5. Conclusions

The highlight of this study is the preparation of the type 2 diabetic gastroparesis rat model, the use of ultrasound technology to assess the gastric emptying of rats, and exploring the effect of EA on gastric antral motility and the ENS in DGP rats. Our study showed that EA at ST36 has a remarkable therapeutic effect on disorders of glucose metabolism and gastric motility in type 2 diabetic gastroparesis rats. In particular, the repair of neurotransmitters and neurons in the ENS is involved in the improvement of EA to gastric motility disorders.

## Figures and Tables

**Table 1:** Sequences of primers used for qRT-PCR.

Gene	Forward sequence	Reverse sequence
Nos1	GACTCACCCCGTCCTTTG	GCCTGCCCCATTAGCTT
ChAT	CTCCCTCAGTGCCAGAAGA	GGGTGGACAACATCAGATCA
Tubb3	CCCAGCGGCAACTATGT	CTCCAGGTCCACCAGAATG

## Data Availability

The data used to support the findings of this study are available from the corresponding author upon request.

## Abbreviations

ACh: Acetylcholine
AUC: Area under curve
ChAT: Choline acetyltransferase
CON: Control
DGP: Diabetic gastroparesis
DM: Diabetic mellitus
EA: Electroacupuncture
ENS: Enteric nervous system
FBG: Fasting blood glucose
GAPDH: Glyceraldehyde-3-phosphate dehydrogenase
HOMA-IR: Homeostatic model assessment of insulin resistance
HRP: Horseradish peroxidase
ICC: Interstitial cells of Cajal
IGTT: Intraperitoneal glucose tolerance test
NFBG: Nonfasting blood glucose
NO: Nitrous oxide
nNOS: Neuronal nitric oxide synthase
PGP9.5: Protein gene product 9.5
PVDF: Polyvinylidene fluoride
qRT-PCR: Quantitative real-time reverse transcription polymerase chain reaction
SD: Sprague-dawley
SP: Substance P
STZ: Streptozotocin
T1DM: Type 1 diabetes mellitus
T2DM: Type 2 diabetes mellitus.
